# Dynamic coding network for robust fruit detection in low-visibility agricultural scenes

**DOI:** 10.3389/fpls.2025.1670790

**Published:** 2025-10-27

**Authors:** Hanyun Lu, Teng Jin, Chen Wan, Shuifa Sun, Xiumei Zhou, Fangyi Wang

**Affiliations:** ^1^ Hubei Key Laboratory of Intelligent Vision Based Monitoring for Hydroelectric Engineering, China Three Gorges University, Yichang, China; ^2^ College of Computer and Information Technology, China Three Gorges University, Yichang, Hubei, China; ^3^ School of Information Science and Technology, Hangzhou Normal University, Hangzhou, Zhejiang, China; ^4^ Innovation and Entrepreneurship College of China Three Gorges University, Yichang, Hubei, China

**Keywords:** fruit detection, low visibility, agricultural scene, dynamic coding, attention mechanisms

## Abstract

**Introduction:**

Accurate fruit detection under low-visibility conditions such as fog, rain, and low illumination is crucial for intelligent orchard management and robotic harvesting. However, most existing detection models experience significant performance degradation in these visually challenging environments.

**Methods:**

This study proposes a modular detection framework named Dynamic Coding Network (DCNet), designed specifically for robust fruit detection in low-visibility agricultural scenes. DCNet comprises four main components: a Dynamic Feature Encoder for adaptive multi-scale feature extraction, a Global Attention Gate for contextual modeling, a Cross-Attention Decoder for fine-grained feature reconstruction, and an Iterative Feature Attention mechanism for progressive feature refinement.

**Results:**

Experiments on the LVScene4K dataset, which contains multiple fruit categories (grape, kiwifruit, orange, pear, pomelo, persimmon, pumpkin, and tomato) under fog, rain, low light, and occlusion conditions, demonstrate that DCNet achieves 86.5% mean average precision and 84.2% intersection over union. Compared with state-of-the-art methods, DCNet improves F1 by 3.4% and IoU by 4.3%, maintaining a real-time inference speed of 28 FPS on an RTX 3090 GPU.

**Discussion:**

The results indicate that DCNet achieves a superior balance between detection accuracy and computational efficiency, making it well-suited for real-time deployment in agricultural robotics. Its modular architecture also facilitates generalization to other crops and complex agricultural environments.

## 1 Introduction

In the agricultural domain, computer vision has been widely applied, particularly in orchard management systems. Modern harvesting robots employ advanced models for classification, detection, and related tasks, enabling efficient yield estimation and crop monitoring Akiva et al. (2020); Sa et al. (2016); Bargoti and Underwood (2017); Guo et al. (2024); Wang et al. (2022); Lehnert et al. (2016); Bac et al. (2017). However, most existing fruit detection approaches are designed for ideal conditions, neglecting weather-affected scenarios that significantly degrade image quality and compromise detection accuracy. The outdoor nature of orchards introduces further challenges for visual systems Kamilaris and Prenafeta-Boldú (2018), as strong sunlight, precipitation, fog, and snow often impair visibility. Dense foliage additionally obscures fruits, creating background noise and visual complexity. Nevertheless, fruit detection under low-visibility outdoor conditions is essential for agricultural object detection, and achieving high accuracy is crucial for smart agriculture.

Existing state-of-the-art models such as YOLOv7/v8, HitNet, and Deformable DETR have demonstrated remarkable performance in general object detection tasks. However, these frameworks are either optimized for high-quality images or rely heavily on anchor-based predictions and large-scale transformer backbones, which tend to degrade under adverse conditions such as fog, rain, or low illumination. In contrast, the proposed DCNet is specifically designed for agricultural environments with low-visibility. Unlike these generic detectors, DCNet integrates four tailored modules—Dynamic Convolution, Global Attention Gate, Cross-Attention Decoder, and Iterative Feature Attention—that collectively enable fine-grained feature recovery and effective noise suppression. This combination systematically addresses visibility-induced feature loss, which has not been explicitly solved in previous fruit detection studies.

Although existing deep learning methods achieve promising results in standard environments, low-visibility conditions in real-world agricultural scenarios—such as insufficient lighting, haze, occlusion, or dense fruit clustering—still significantly degrade detection performance. The main technical gaps in current approaches are as follows:

- Feature extraction under low-visibility conditions is not robust, often leading to missed detections or false positives.
- Existing feature fusion strategies inadequately integrate global and local information, limiting the utilization of multi-scale features in complex scenes.
- There is a lack of iterative feature optimization mechanisms tailored for low-visibility conditions, resulting in suboptimal object localization.
- Most studies lack systematic approaches to improve model generalization across diverse complex environments.

To address these issues, this study proposes a robust fruit detection framework that maintains high detection accuracy and instance completeness across diverse complex environments, providing technical support for intelligent monitoring in real-world orchard scenarios.

The main contributions of this work are summarized as follows:

- We propose DCNet, a novel modular detection framework specifically designed to address low-visibility challenges in agricultural scenarios.
- Our method incorporates multi-scale dynamic encoding for robust feature extraction under visibility noise, enhancing the representation of occluded or poorly illuminated fruits.
- Innovative feature fusion strategies are developed, integrating global attention, local attention, and cross-attention mechanisms to improve detection accuracy.
- An iterative feature fusion mechanism is employed to optimize feature representation, significantly enhancing object localization performance in challenging low-visibility conditions.

The remainder of this paper is organized as follows: section 2 introduced the existing methods and related research; Section 3 presents the proposed DCNet framework and its main components; Section 4 reports the experimental setup, results, and ablation studies; Section 5 provides further analysis and discussion of the findings; Section 6 concludes the paper and outlines directions for future research.

## 2 Related work

Recent years have witnessed remarkable progress in deep learning-based object detection algorithms, which have progressively supplanted conventional approaches. Contemporary detection methodologies can be categorized principally into three paradigms: anchor-based two-stage methods, anchor-based single-stage methods, and anchor-free algorithms Zand et al. (2021); Cheng et al. (2022); Wang et al. (2020).

Single-stage detectors, such as YOLOv7 Wang et al. (2023) and Dynamic YOLO Li et al. (2019), perform object localization and classification in a unified framework by directly predicting bounding boxes and class probabilities on a dense grid, without the need for region proposals. This design confers notable computational efficiency; however, it may compromise detection accuracy, particularly for small or densely packed objects, due to the limitations imposed by fixed-grid anchors. In contrast, two-stage approaches, including R-CNN Girshick et al. (2014), Fast R-CNN Girshick (2015), and more recent frameworks Kang et al. (2024), decompose the detection task into a region proposal stage followed by region-wise classification and bounding box refinement. By leveraging hierarchical feature representations and region-specific processing, these methods achieve superior accuracy, albeit at the cost of increased computational complexity.

Traditional object detectors can usually achieve satisfactory performance, but most object detection models are carried out under ideal preconditions, such as high-quality images, which limits their application in practice. In cases of degraded image quality, their effectiveness may be significantly compromised. For instance, in outdoor object detection tasks, weather conditions are key factors that significantly affect the performance of detection models. Under severe weather conditions (e.g., precipitation, fog, or snow), both human visual perception and vision-based detection systems Munir et al. (2024); Li et al. (2018a) experience significant performance degradation Bac et al. (2017); Lehnert et al. (2016). To address these weather-induced challenges, substantial research efforts have yielded multiple solution strategies, broadly classifiable into four categories: image enhancement-based strategy Li et al. (2018b); Chen et al. (2019), prior knowledge-integrated strategy Liu et al. (2022); Li et al. (2017); Huang et al. (2020), unsupervised learning Chen et al. (2021); Sindagi et al. (2020); Zhu et al. (2017); Wang et al. (2004) and knowledge distillation-based multi-task learning methods Yang et al. (2022).

These methods aim to simultaneously learn feature representations under various weather conditions Zhang and Patel (2018). Although the aforementioned methods have made progress in improving object detection performance under harsh weather conditions, several challenges remain. First, most existing methods are optimized for specific weather conditions, making it difficult to handle complex and dynamic real-world environments. Second, many of these approaches require additional data collection and annotation, which increases the cost of system deployment.

In this paper, we proposes a novel modular framework specifically designed to address low-visibility detection challenges, aiming to mitigate performance degradation caused by visibility noise while establishing benchmark standards for agricultural applications. Through comprehensive analysis of low-visibility noise interference mechanisms in feature extraction, we introduce DCNet: a Dynamic Coding-based detection network for agricultural scenarios. Our approach incorporates multi-scale dynamic encoding for robust feature extraction, develops innovative fusion strategies for global and local features, and proposes an advanced decoder architecture integrating global attention, local attention, and cross-attention mechanisms. Furthermore, an iterative feature fusion mechanism is incorporated to optimize feature representation, significantly enhancing object localization accuracy in challenging visibility conditions. Our proposed method achieves effective fruit detection in challenging scenes, providing a crucial basis for orchard management in low-visibility environments.

## 3 Methods

**Figure 1:**
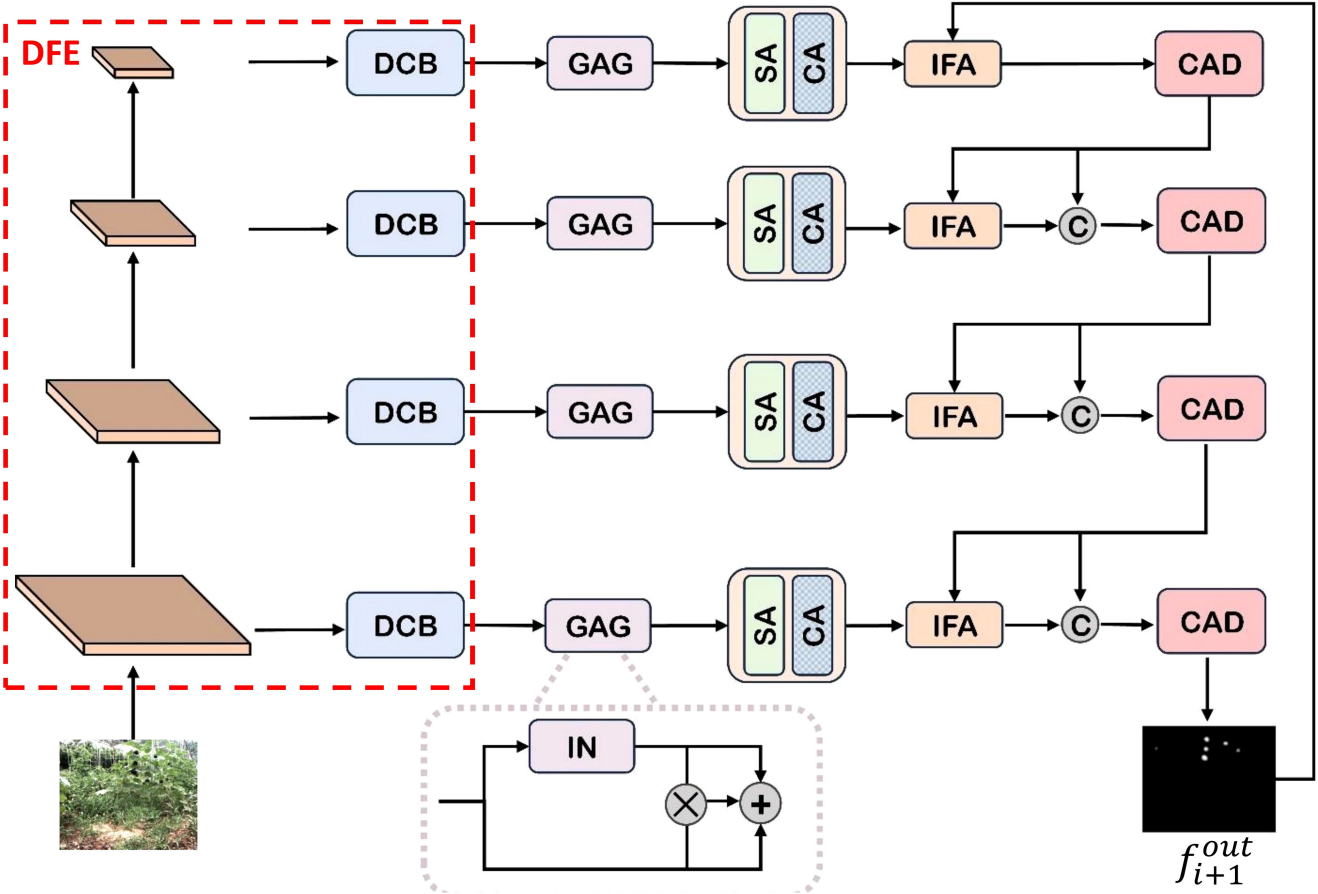
Architecture overview. DFE employs a visual pyramid to extract image features and generate multi-scale feature maps. It effectively retains fine-grained local information while fusing multi-layer semantic features of the object in a coherent manner. GAG leverages the attention mechanism to dynamically mitigate interference caused by low-visibility and enhances the response within the object region. CAD utilizes the cross-attention mechanism to capture long-range dependencies, thereby achieving robust multi-scale information fusion. IFA facilitates cross-layer interaction between high-level semantic features and low-level fine-grained features via an iterative optimization mechanism, with a focus on refining representations in the object area.

We present a novel unimodal object detection framework, DCNet as illustrated in **Figure 1**, our comprehensive framework comprises four key components: (1) a dynamic feature encoder (DFE) for adaptive feature representation, (2) a global attention gate (GAG) for contextual modeling, (3) a cross attention decoder (CAD) for discriminative feature reconstruction, and (4) an iterative feature attention (IFA) mechanism for progressive feature refinement.

### 3.1 Dataset description

LVScene4K Dataset. The LVScene4K dataset, constructed for this study, was collected at Huazhong Agricultural University. Images were captured outdoors using a Hikvision industrial camera by rotating around each crop at fixed angles, thus simulating realistic orchard conditions from multiple viewpoints. The dataset covers diverse fruit categories, including grapes, kiwi, oranges, pears, pomelos, persimmons, pumpkins, and tomatoes. For each crop type, between 100 and 300 images were collected, resulting in a total of approximately 4,000 images. All images were acquired under natural outdoor conditions with varying illumination and partial occlusions, reflecting real-world agricultural challenges. The final dataset contains images with a resolution of 704 × 704 pixels.

Annotation. A two-stage annotation procedure was employed. First, bounding boxes for fruits were manually annotated using LabelImg. Subsequently, pixel-level instance masks were generated by a professional annotation service provider to ensure high-quality segmentation. This dual-annotation approach enables the dataset to be applicable both for detection and segmentation tasks.

Environmental conditions. Our dataset covers images collected outdoors under different daylight conditions (noon and dusk). To further verify the reliability of the proposed method under various low-visibility scenarios, we employed the augimg toolkit to perform randomized data augmentation. Rain, snow, and fog—each divided into three intensity levels—were randomly superimposed onto the images. For instance, when “fog” is selected, the image may receive light fog, normal fog, or heavy fog. Although the augmentation is stochastic, all modifications obey basic natural laws: if an image already contains over-exposure, fog will not be added, because strong sunlight and fog rarely coexist. Following this protocol, every augmented image carries 1–3 simultaneous low-visibility factors, yielding 20 distinct combinations of adverse conditions in total. After augmentation, these weather-corrupted images are labeled as the “hard” subset, which is used to examine the model’s robustness under extremely challenging visibility conditions.

### 3.2 Dynamic feature encoder

#### 3.2.1 Multi-scale feature encoder

The Transformer architecture has achieved remarkable success in natural language processing (NLP) and has been progressively extended to the computer vision domain, demonstrating considerable potential in image classification tasks. The vision transformer (ViT) partitions an input image into a series of non-overlapping patches, which are subsequently processed as sequential tokens, thereby successfully adapting the Transformer framework to visual tasks while achieving performance on par with traditional convolutional neural networks (CNNs).

However, despite its superior performance in classification tasks, ViT exhibits certain limitations in scenarios requiring multi-scale feature representation. In contrast, the pyramid vision transformer (PVT) Wang et al. (2021), which incorporates a hierarchical feature pyramid structure, demonstrates exceptional capability in computer vision tasks such as object detection. This enhanced performance primarily stems from its effective exploitation of multi-scale features during both the encoding and decoding phases.

During the encoding stage, PVT employs a feature pyramid architecture to extract multi-scale object representations from the input image. This hierarchical encoding strategy progressively reduces feature resolution while integrating the spatial reduction attention (SRA) mechanism, which substantially mitigates computational overhead. Consequently, the model maintains high efficiency even under constrained computational resources, making it particularly suitable for large-scale vision applications.

#### 3.2.2 Dynamic convolution module

Under low-visibility conditions, environmental perception is significantly impaired due to reduced visibility and substantial variations in light intensity, which severely degrade image quality and pose considerable challenges for object detection models. Notably, feature importance exhibits spatial heterogeneity across image regions. For images affected by adverse lighting conditions or degraded by atmospheric noise, traditional convolutional operations may fail to capture subtle feature representations. To address this limitation, we propose the implementation of adaptive convolutional kernels with dynamic configurations, including variable kernel sizes, shapes, and weighted convolution outputs. Drawing upon this conceptual framework, we introduce a DCB module (Dynamic Convolution Module) Chen et al. (2020). Dynamic convolution allows for more detailed extraction of local features in an image, which is especially crucial under low-visibility conditions. By adjusting the convolution kernels, dynamic convolution captures key information in the image at a finer granularity, such as object contours and textures. The dynamic convolution module calculates weights and flexibly selects appropriate kernels to handle images with significant differences, optimizing for low-visibility environmental conditions. This approach helps the model accurately locate and recognize objects, enabling better adaptation to such environments and enhancing the precision of image feature extraction, thus improving the model’s ability to accurately identify objects.

**Figure 2:**
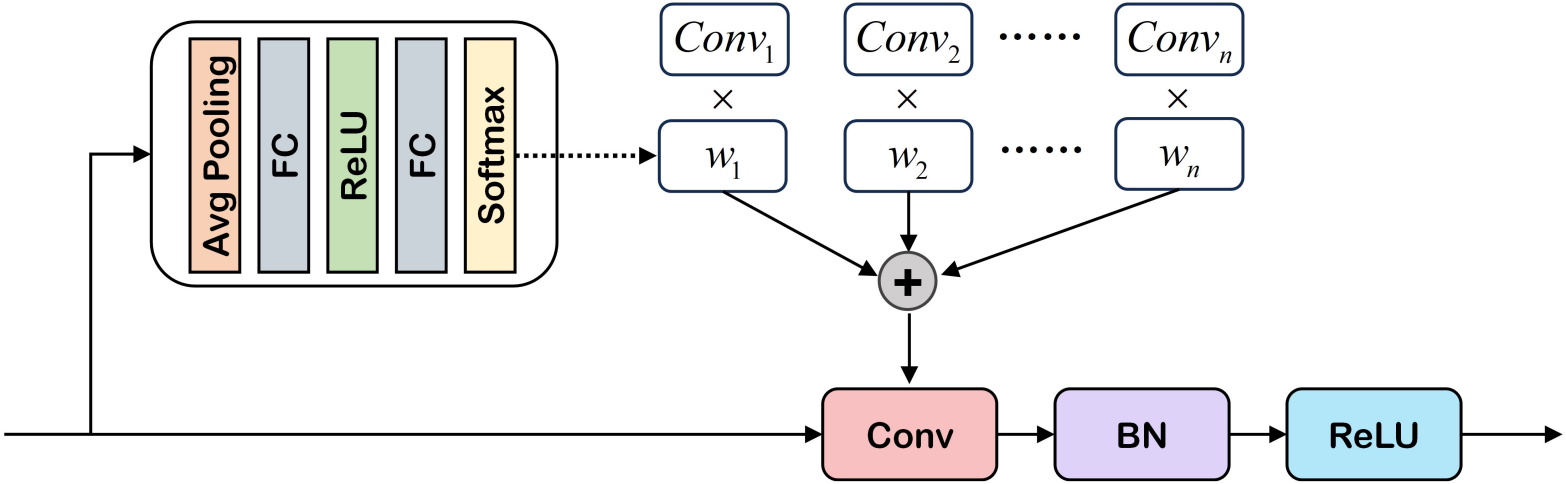
Illustration of dynamic feature encoder. DFE consists of two parts: PVT (obtaining multi-scale features) and DCB (enhancing the ability to extract local features in low-visibility areas). For more details. Please refer to § 3.2.1 and § 3.2.2 for details.

As illustrated in **Figure 2**, the DCB module processes each input feature map ${\left\{f_{n}\right\}}_{n-1}^{4}\in {\mathbb{R}}^{B\times C\times \frac{H}{2^{n+1}}\times \frac{N^{N}}{2^{n+1}}}$ through a cascaded architecture comprising adaptive pooling layers, fully connected layers, and ReLU activation functions to compute attention weights. These weights dynamically modulate both the convolutional kernel dimensions and operation weights. The final convolutional output is generated through a weighted fusion of multiple convolution results, followed by batch normalization (BN) and ReLU activation. The resultant feature representation after dynamic convolution is denoted as ${\left\{f_{n}\right\}}_{n-1}^{4}\in {\mathbb{R}}^{B\times C\times \frac{H}{2^{n+1}}\times \frac{W}{2^{n+1}}}$ .

### 3.3 Global attention gate

When confronted with low-visibility images, the model’s ability to discriminate fruit objects diminishes due to a decline in image quality. To address this challenge, we propose a learnable adaptive GAG, a parameterizable normalization framework that enables the model to autonomously learn noise characteristics induced by adverse visibility conditions (e.g., low contrast and haze occlusion) during feature decoding. This mechanism facilitates adaptive parameter adjustment to enhance object discrimination performance.

Specifically, GAG architecture first applies instance normalization to input features, followed by a sophisticated linear combination operation between the original input and normalized features. This process involves multiplying the original input by the normalized result, and then element-wise adding the product to both the normalized result and the original input. Twofold benefits are achieved through this design: (1) effective learning of adaptive normalized features while (2) preserving crucial details from the original input, thereby preventing feature representation bias and alleviating overfitting tendencies. The mathematical formulation of GAG is expressed as Equation (1):


(1) $$IN(f_{n})=\eta (\frac{f_{n}-\mu (f_{n})}{\sigma (f_{n})})+\epsilon$$

(2) $$f_{n}^{i}=f_{n}⊕IN(f_{n})⊕IN(f_{n})⊗f_{n}$$

Where IN(·) denotes instance normalization, η and ϵ represent learnable parameters, μ(·) and σ(·) indicate mean and variance operations, respectively, ⊕ and ⊗ correspond to Equation (2) element-wise addition and multiplication, and ${\left\{f_{n}^{i}\right\}}_{n=1}^{4}\in {\mathbb{R}}^{B\times C\times \frac{H}{2^{n+1}}\times \frac{W}{2^{n+1}}}$ signifies the final output of combined features. To further enhance object-background discrimination capability under compromised visibility conditions and mitigate the obstructive effects of precipitation elements, we incorporate a dual-attention mechanism. First, a spatial attention module processes the features to emphasize location-specific information. Recognizing that different channel features exhibit varying importance under distinct noise conditions, we subsequently employ a channel attention mechanism to dynamically recalibrate channel-wise feature weights during decoding. This ensures optimal utilization of noise-specific discriminative features. The complete attention process is formally defined as Equations (3):


(3) $$f_{n}^{f}=f_{n}^{i}⊗CA(f_{n}^{i})⊗SA(f_{n}^{i}⊗CA(f_{n}^{i}))$$

Where CA(·) and SA(·) represent spatial and channel attention operations respectively, ⊗ denotes element-wise multiplication, and $f_{n}^{f}$ denotes the final attended feature map.

The GAG module can be thought of as a two-step “adaptive lens cleaning” process for fruit detection. First, the normalization step works like automatically adjusting the brightness and contrast of an image to counteract fog, shadows, or dim lighting. This ensures that the model sees a clearer, more standardized version of the fruit features without losing the original details. Second, the attention mechanism acts like focusing your eyes: spatial attention highlights “where” the fruits are in the image (locations), while channel attention emphasizes “what kind of details” are most useful (such as color, texture, or shape under noisy conditions). Together, these steps allow the model to suppress irrelevant noise while preserving and enhancing fruit-specific characteristics, just as a human observer would adjust their vision when looking at a fruit tree in foggy or rainy conditions.

### 3.4 Cross-attention-based decoder

#### 3.4.1 Cross-attention block

**Figure 3:**
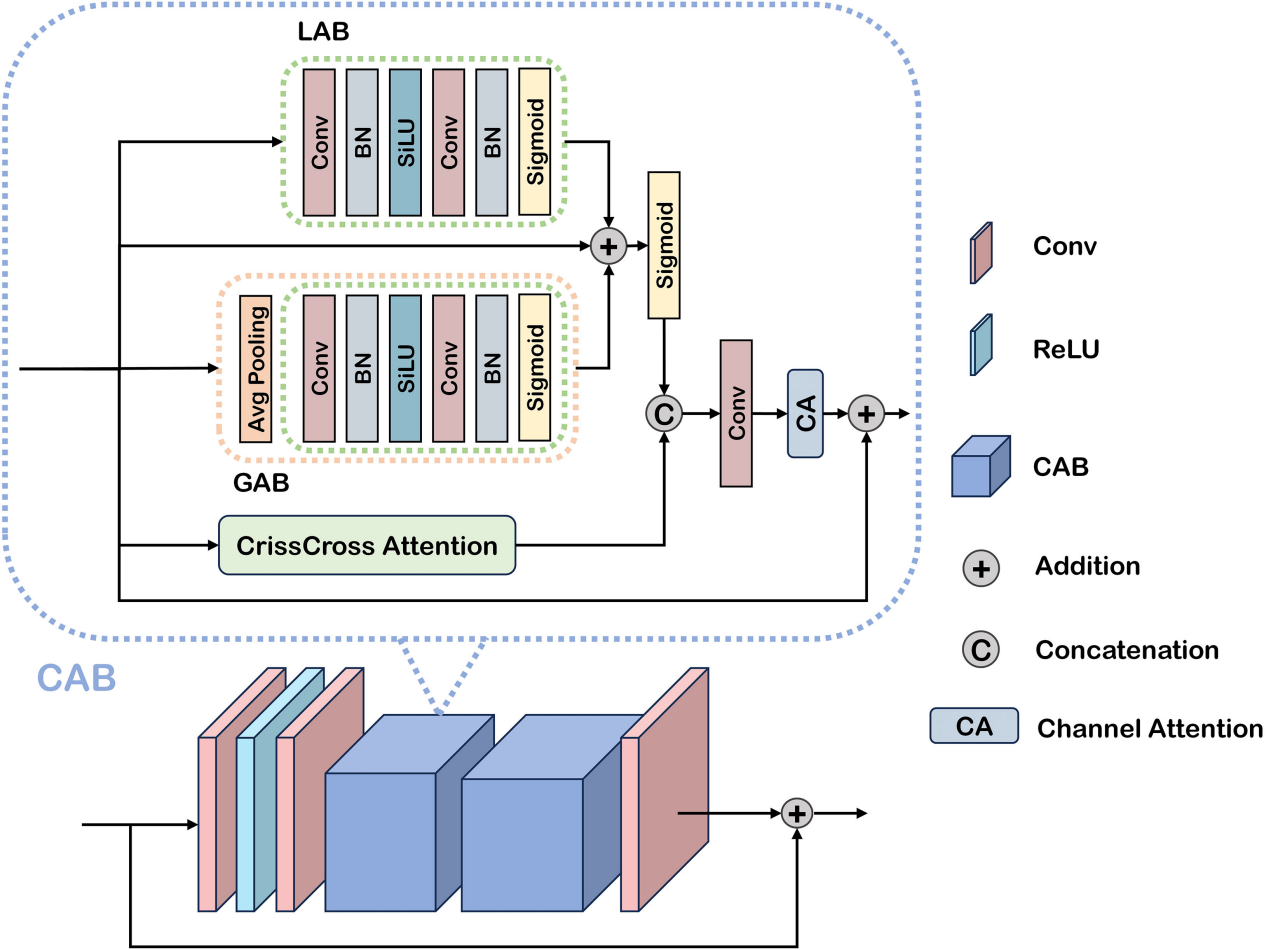
Illustration of the cross-attention-based decoder.


Agricultural scenes with limited visibility present particularly challenging backgrounds, demanding heightened attention to detail for effective object detection. To address this characteristic, we propose a dual attention mechanism comprising local attention block (LAB) and global attention block (GAB) as illustrated in **Figure 3**, designed to capture both localized and comprehensive feature representations, respectively. Specifically, we introduce a Cross Attention Block that performs cross-attention computations along both horizontal and vertical orientations. This design enhances the model’s capacity for multi-directional and multi-scale perception, facilitating the effective fusion of spatial information across different scales. By capturing the intricate relationships between fruit objects and their surrounding environment, the CAB enables dynamic adjustment of attention based on regional feature distributions, thereby mitigating the adverse effects of poor weather conditions on detection performance. CAB can be expressed as:

(4) $$LAB(f_{n}^{t})=\text{Sigmoid}(CBSCB(f_{n}^{t}))$$

(5) $$GAB(f_{n}^{t})=\text{AvgPooling}(LAB(f_{n}^{t}))$$

(6) $$f_{n}^{LG}=\text{Sigmoid}(LAB(f_{n}^{t})+GAB(f_{n}^{t})+f_{n}^{t})$$

(7) $$f_{n}^{cab}=CA(\text{Conv}(\text{Concat}(f_{n}^{LG},CCA(f_{n}^{t}))))+f_{n}^{t}$$

Where LAB(·) Equation (4) denotes the Local Attention Block, ${\left\{f_{n}^{f}\right\}}_{n=1}^{4}$ denotes the feature input to the CAB, CBSCB(·) refers to the cascaded “Conv-BN-SiLU-Conv-BN” layers, GAB(·) Equation (5) represents the Global Attention Block, AvgPooling(·) indicates adaptive average pooling Equation (6), ${\left\{f_{n}^{LG}\right\}}_{n=1}^{4}$ refers to the fused feature representation of local and global features, ${\left\{f_{n}^{cab}\right\}}_{n=1}^{4}$ denotes the feature output of the CAB, CA(·) represents channel attention, Conv(·) refers to the convolutional layer, Concat(·) Equation (7) denotes concatenation along the channel dimension, and CCA(·) represents cross attention.

Within our framework, the CAD integrates convolutional layers, ReLU activation functions, and two sequentially stacked CABS. This architecture progressively refines feature extraction in low-visibility agricultural scenes, with the first CAB capturing local and global features and the second CAB further enhancing their integration. Through this cascaded attention mechanism, the model achieves improved recognition of subtle object characteristics under challenging conditions, including low contrast, high noise, and detail degradation, ultimately leading to superior detection accuracy. The decoding process of CAD is formulated as Equations (8):


(8) $$f_{n}^{c}=Conv(CAB(CAB(CAB(((CRC(f_{n}^{f}))))))$$

The CAB can be viewed as a “discussion process” between different perspectives of the same fruit image. The LAB acts like zooming in with a magnifying glass, focusing on fine-grained details such as fruit edges or small color changes. The GAB works like stepping back and looking at the entire tree, capturing overall context such as clusters of fruits or background distribution. By combining these two perspectives, the model learns both “the details” and “the big picture.” - Finally, the channel and coordinate attentions act like specialists who decide which type of information (e.g., color, texture, spatial position) is most trustworthy under current conditions.

Together, this cross-attention process enables the network to integrate detailed local information with global context, ensuring more accurate fruit detection even in cluttered orchard environments.

#### 3.4.2 Iterative feature attention

Following the CAD processing, each feature level generates a corresponding coarse prediction map. These hierarchical predictions exhibit distinct characteristics: higher-level features encode more abstract semantic information, while lower-level features preserve fundamental structural details such as shape and texture. To effectively leverage this complementary information, the coarse prediction map from higher level features is concatenated with the subsequent level’s features, ensuring comprehensive utilization of both semantic and low-level visual cues.

**Figure 4:**
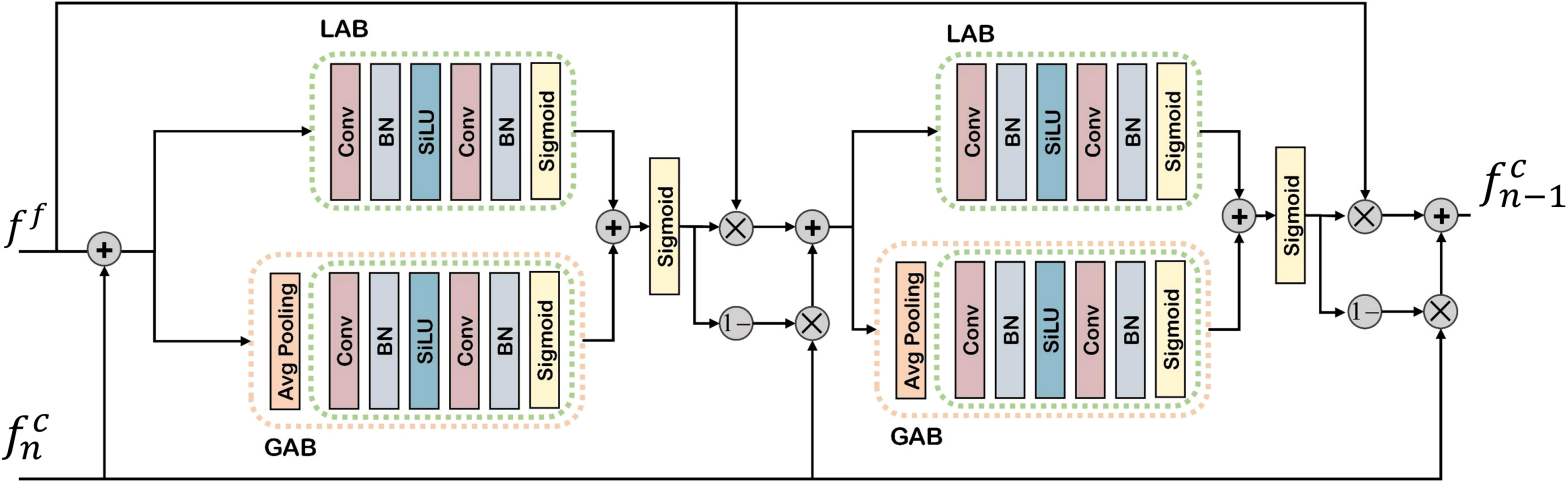
Illustration of the proposed iterative feature attention. Details can be observed more clearly upon zooming in.


As illustrated in **Figure 4**, drawing inspiration from Dai et al. (2021), we propose an IFA mechanism to systematically explore the relationship between coarse prediction maps and adjacent feature levels. The IFA operates across both local and global scopes, dynamically computing attention weights based on the preceding level’s prediction map and the current feature level. This process directs the decoder’s focus toward object regions, thereby enhancing the model’s discriminative capability. The IFA mechanism is formally expressed as:

(9) $$\alpha_{1}=\text{Sigmoid}(LAB(f_{n}^{c}+f^{f})+GAB(f_{n}^{c}+f^{f}))$$

(10) $$f_{n}^{c1}=(1-\alpha_{1})\times f_{n}^{c}+\times \alpha_{1}\times f^{f}$$

(11) $$\alpha_{2}=\text{Sigmoid}(LAB(f_{n}^{c1}+GAB(f_{n}^{c1}))$$

(12) $$f_{n-1}^{c}=(1-\alpha_{2})\times f^{f}+(1-\alpha_{2})\times f_{n}^{c}$$

Where $\alpha_{1}$ Equation (9) and $\alpha_{2}$ Equation (11) represent the attention values obtained from two separate computations, Sigmoid(·) denotes the sigmoid function, LAB(·) refers to the local attention module, GAB(·) represents the global attention module, ${\left\{f_{n}^{c}\right\}}_{n=2}^{4}$ indicates the coarse prediction map of the decoder, ${\left\{f_{n-1}^{c}\right\}}_{n=2}^{4}$ Equation (12) denotes the features of the next level, and ${\left\{f_{n}^{c1}\right\}}_{n=1}^{4}$ Equation (10) represents the transitional features between the two attention parts before and after IFA.

Beyond intra-level feature refinement, the IFA mechanism is extended to operate across multiple iteration rounds. Starting from the second iteration, features are progressively updated by incorporating attention guided information from previous iterations. This cross-iteration refinement is formulated as:


(13) $$f_{4}^{i+1,\text{ifa}}=\text{IFA}(f_{4}^{i+1,f},f_{4}^{i,\text{pre}})$$

Where $f_{4}^{i+1,\text{ifa}}$ Equation (13) denotes the feature updated after the i+1 -th iteration of the 4th-level feature, $f_{4}^{i+1,f}$ represents the feature before the i+1 -th iteration update of the 4th-level feature, $f_{4}^{i,\text{pre}}$ indicates the final prediction map after the i+1 -th iteration, and IFA(·) refers to the Iterative Feature Attention.

Upon completing the final iteration, to preserve critical low-level details (e.g., edges and textures), the optimized high-level feature $f_{2}^{c}$ is upsampled and fused with the lowest-level feature $f_{1}^{f}$ . The concatenated features are then processed by a Multi-scale Convolution Module (MC), which employs parallel convolutional layers of varying receptive fields to capture discriminative patterns at multiple scales. The final prediction $F^{\text{pre}}$ is derived as:


(14) $$F_{f}^{\text{pre}}=MC(\text{Concat}(f_{1}^{f},\text{up}(f_{2}^{c})))$$

Where MC(·) Equation (14) denotes the multi-scale convolutional fusion module, up(·) represents the upsampling operation.

The IFA module can be thought of as a “step-by-step polishing process”: Imagine restoring an old photograph. The first pass removes the obvious scratches, but some finer details are still unclear. On the second pass, the image is enhanced further, focusing on regions that were previously overlooked. Each new iteration builds upon the improvements of the last, ensuring that both fine-grained details and overall clarity are progressively optimized.

In the context of fruit detection, IFA repeatedly “revisits” the intermediate feature maps, strengthening useful patterns (e.g., fruit boundaries, texture cues) while filtering out background noise (e.g., leaves, branches). This iterative refinement leads to more robust and accurate detection results under challenging visibility conditions.

#### 3.4.3 Loss function

In the decoding phase of our proposed method, we employ an iterative attention mechanism for progressive feature refinement, where each iteration generates an intermediate feature map. To ensure comprehensive supervision throughout this hierarchical optimization process, our loss function incorporates both the intermediate outputs from each iteration ${\left\{f^{i,\text{pre}}\right\}}_{i=1}^{3}$ and the final prediction map $F_{f}^{\text{pre}}$ . Specifically, we formulate a multi-level supervision strategy where each iterative prediction is constrained by the loss function to maintain optimization consistency.

To effectively quantify the prediction errors and enhance the model’s capability in detecting challenging objects, we utilize a weighted binary cross-entropy loss $L_{BCE}^{\omega }$ and a weighted intersection-over-union loss $L_{IoU}^{\omega }$ to supervise both intermediate and final predictions. These loss functions are designed to penalize misclassifications while emphasizing hard samples, thereby improving model robustness. The overall prediction loss is denoted as $L_{\text{stage}}=L_{\text{BCE}}^{\omega }+L_{\text{IoU}}^{\omega }$ Equation (15).

Furthermore, to account for the varying reliability of coarse predictions at different iterations, we introduce a weight parameter *ℓ* to dynamically adjust the contribution of each intermediate prediction, ensuring that earlier coarse predictions contribute adaptively to the overall optimization process. The final loss function is formally defined as:


(15) $$L=L_{\text{stage}}(F_{f}^{\text{pre}},GT)+\sum_{i=1}^{3}(\ell \times i)(L_{\text{stage}}(f^{i,\text{pre}},GT))$$

## 4 Experimental results

### 4.1 Experimental settings

Current approaches for enhancing detection performance under low-visibility conditions typically involve reconstructing high-quality images from degraded inputs prior to detection. Following this paradigm, the present study systematically evaluates the efficacy of the proposed method through comprehensive experiments on both “simple” and “challenging” samples from the LVScene4K dataset. For benchmarking purposes, we employ HITNet Hu et al. (2023) as our baseline detection framework, representing the current state-of-the-art (SOTA) for normal visibility conditions. To establish performance comparisons, we integrate existing SOTA image denoising models with this baseline, constructing a two-stage object detection pipeline incorporating a denoising module. Furthermore, we implement comparative evaluations using a recently proposed SOTA semantic segmentation network specifically optimized for adverse weather conditions, which performs binary segmentation on our dataset. All comparative results are rigorously analyzed relative to the performance of our proposed DCNet.

In this study, Low-Visibility Condition Detection (LVCD) is formulated as a binary segmentation task, wherein the model produces a binarized mask upon object identification. Accordingly, we adopt two well-established region-based evaluation metrics commonly used in segmentation tasks: Intersection over Union (IoU) and the *F*_1_ score. These metrics quantitatively assess the spatial agreement between the model’s predictions and the ground truth annotations, providing robust performance evaluation.

DCNet maintains a moderate level of model complexity. While the introduction of dynamic convolution and attention modules increases the number of parameters and computational cost compared with lightweight detectors, the overall scale of DCNet remains significantly smaller than that of large two-stage detectors such as Mask R-CNN. When evaluated with an input size of 704 × 704 on an NVIDIA RTX 3090 GPU, DCNet achieves an inference speed of approximately 20–30 FPS, which is sufficient for real-time deployment in agricultural robotic systems. During inference, the memory consumption remains below 4 GB, indicating that the proposed network balances accuracy and efficiency effectively, and can be adapted for practical field applications with potential for further optimization on embedded platforms.

DCNet introduces only 8.7% more FLOPs than HitNet while improving IoU by 3.4% (**Table 1**). On Jetson Nano, the edge FPS reaches 11, meeting real-time demand for agricultural robots (≥10 FPS). At the same time, we also evaluated the system’s resource consumption and stability. In the low-load mode, the device’s CPU and GPU usage were low, with a power consumption of 5W, memory usage around 2GB, and the system temperature remained around 45°C, ensuring stability during prolonged operation. In the high-load mode, although the frame rate decreased, the power consumption increased to 10W, and the temperature reached 65°C, the system continued to run stably without crashes or significant performance fluctuations. These results verify that DCNet achieves a favorable trade-off between accuracy and efficiency.

**Table 1:** Performance comparison of different methods on “simple” and “difficult” samples.

Model	Publications	“simple” samples		“difficult” samples	
		IoU	*F*_1_	IoU	*F*_1_
A-HITNet	AAAI23	0.764	0.618	0.726	0.570
B1-FFA	AAAI20	0.743	0.634	0.739	0.586
B2-HDCWNet	ICCV21	0.777	0.635	0.745	0.594
B3-CKT	CVPR22	0.778	0.636	0.750	0.601
C1-Refign	WACV23	0.759	0.656	0.721	0.574
C2-MIC	CVPR23	0.769	0.639	0.749	0.596
C3-CMDA	ICCV23	0.770	0.625	0.749	0.590
DCNet(Ours)		**0.785**	**0.646**	**0.760**	**0.613**

Bold values indicate the best-performing results in the corresponding metric.

### 4.2 Experimental details

The method proposed in this paper is implemented using PyTorch, and all experiments are conducted on an RTX 3090 GPU, with the AdamW optimizer selected for parameter optimization. During the training phase, the total number of epochs is set to 150, with a batch size of 8. Experimental evidence indicates that higher resolution has a positive impact on detection performance, and the study uses a resolution of 704 × 704 as input during both training and testing phases. The learning rate is initialized to 0.0001 and is reduced to one-tenth of its value every 50 epochs. Regarding the optimal number of iterations, we devised a hyper-parameter tuning scheme to secure peak model performance and avoid degradation caused by under- or over-fitting. On both “easy” and “hard” subsets we varied the iteration count and compared the final detection results. As **Table 2** shows, raising the iterations first boosts the F1, but once the number is too large the model begins to over-fit and the F1 drops. Consequently, the tuning experiment identifies 3 iterations as the best setting, and the iterative loss parameter set to 0.2.Wang et al. (2024).

**Table 2:** Results of the experiment on the optimal number of iterations.

Sample	*F*_1_	Iteration				
		1	2	3	4	5
Easy	Score	0.720	0.781	**0.785**	0.777	0.768
Hard	Score	0.691	0.759	**0.760**	0.757	0.725

Bold values indicate the best-performing results in the corresponding metric.

For reproducibility, we provide additional implementation details of DCNet. All input images are resized to 704 × 704 before being fed into the network. Most convolutional layers adopt a kernel size of 3 × 3, while 1 × 1 kernels are used for channel reduction and fusion operations. Stride settings follow common practice: the backbone (PVT-v2) applies stride-2 convolutions for downsampling at multiple stages, whereas the dynamic convolution layers and refinement modules employ stride-1 to preserve spatial resolution. Regarding nonlinear activation, the network mainly uses ReLU and PReLU after convolution operations, SiLU in iterative attention modules, and Sigmoid functions to generate attention weights in channel and spatial attention submodules. This combination ensures both stable training and expressive feature representation.

### 4.3 Experimental analysis

#### 4.3.1 Quantitative analysis

Following the experimental design, this section compares the performance of the basic detection method (A), the integrated denoising-based detection method (B), and the semantic segmentation method tailored for adverse weather conditions (C). To ensure fairness in the comparison of experimental results, all methods used for comparison followed the default settings for model training and evaluation. As shown in **Table 1**, using IoU and F1 score as evaluation metrics, the proposed method consistently outperforms in fruit detection tasks represented by both “simple” and “difficult” samples from the LVCD task, showing significant performance improvements in low-visibility fruit detection compared to other methods. We designs suitable encoding and decoding methods for low-visibility conditions, achieving a notable performance boost over the best object detection and combined detection methods. Additionally, feature optimization and iterative methods tailored for agricultural scenes provide more accurate segmentation results compared to low-visibility segmentation methods applied in other contexts. Furthermore, the proposed method exhibits minimal performance degradation from simple to challenging samples, demonstrating the robustness of the model under various conditions.

**Figure 5:**
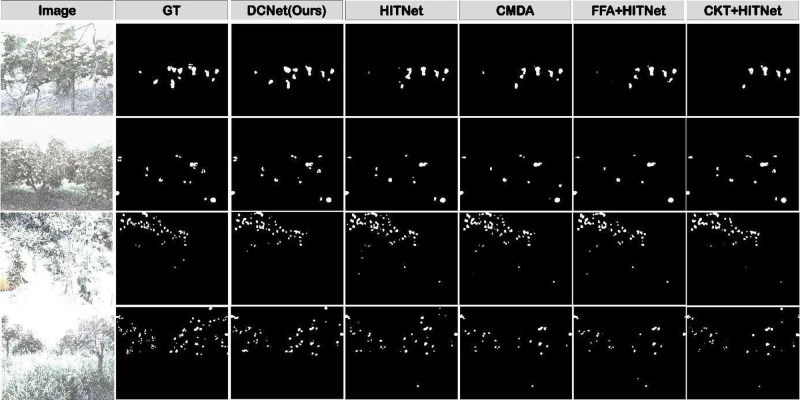
Qualitative comparison of fruit detection results under low-visibility conditions. White regions indicate predicted fruit masks, while black regions denote background.

This study employs qualitative experiments to assess the performance of models in low-visibility scenes. As demonstrated in **Figure 5**, a comprehensive comparative evaluation was conducted between the proposed methodology and multiple baseline approaches. In comparison with the baseline HitNet, our proposed DCNet achieves substantial enhancements in detection accuracy, significantly mitigating the occurrence of both false detections and missed detections. The visualization results indicate that integrating HitNet with various denoising models leads to noticeable performance improvements compared to using HitNet alone. This enhancement indirectly verifies the effectiveness of the denoising module in low-visibility environments.

While existing semantic segmentation approaches tailored for low-visibility conditions have shown promising performance, their generalized design often constrains their applicability in the intricate contexts of agricultural environments. In contrast, the method proposed in this study achieves a marked reduction in false positives and demonstrates robust performance in accurately detecting fruit targets, even under severe visual degradation. As evidenced by the comparative results, our approach consistently surpasses traditional unimodal methods, underscoring its enhanced adaptability and efficacy in handling the challenging visibility conditions frequently encountered in agricultural scenes.

#### 4.3.2 Ablation study

To investigate the effectiveness of the DCNet network, we conducted ablation studies on each of the introduced modules using the LVScene4K dataset. The detailed results are presented in **Table 3**.

**Table 3:** Ablation study of our DCNet. “w/o” indicates that this module is removed.

Model	“simple” samples		“difficult” samples	
	IoU *F*_1_	IoU *F*_1_	IoU *F*_1_	IoU *F*_1_
w/o DFE	0.767	0.622	0.749	0.599
w/o GAG	0.750	0.601	0.710	0.551
w/o CAD	0.742	0.590	0.735	0.581
w/o IFA	0.768	0.623	0.756	0.607
DCNet	**0.785**	**0.646**	**0.760**	**0.613**

Bold values indicate the best-performing results in the corresponding metric.

As shown in **Table 3**, regardless of which module is removed, the model’s performance degrades on both simple and difficult samples. Among the difficult samples, the removal of the GAG module leads to the most significant decline in performance. This indirectly demonstrates that GAG effectively mitigates interference caused by low-visibility and enhances the model’s focus on object regions. For simple samples, CAD has the most substantial impact on the results, highlighting its mechanism of fusing local and global features for accurately locating objects in complex scenarios, thereby significantly improving the detection of fruit objects under conditions of low-visibility.

## 5 Discussion

To align more closely with the difficulties and challenges present in real-world agricultural scenarios, this study conducted an effectiveness evaluation on the LVScene4K dataset specifically designed for low-visibility object detection. This dataset includes low-visibility images caused by outdoor factors such as exposure, rain, snow, and fog. Experimental results demonstrate that the proposed DCNet surpasses the baseline model in both detection accuracy and robustness, particularly in challenging samples.

Through an in-depth analysis of the roles of each component of the model, it was found that the improved global attention mechanism GAG significantly enhances the detection effect of objects under low-visibility conditions, especially regarding performance on difficult samples within the dataset. Compared to the missed detections and false positives observed in the previous object detection model HitNet, the proposed DCNet markedly enhances object feature representation and improves the model’s noise suppression capability in low-visibility scenes by refining the global attention mechanism. The model’s superior performance under low-visibility provides strong support for its practical application.

Considering the practical application requirements, where both detection accuracy and real-time performance are critical, subsequent research will focus on enhancing the model’s real-time performance by reducing its scale and increasing detection speed, ensuring it better meets actual needs and contributes to fruit object detection in low-visibility agricultural scenarios.

Furthermore, most existing agricultural object detection studies are conducted under ideal conditions, assuming high-quality images. However, detection under low-visibility remains relatively unexplored. Although this study has analyzed low-visibility conditions in detail, it is acknowledged that real-world agricultural scenarios often present additional challenges beyond those considered here. Future research will aim to enrich the understanding of difficulties and challenges brought about by diverse outdoor conditions to fruit object detection.

While all experiments in this study were conducted on the LVScene4K dataset, the proposed DCNet framework is not dataset-specific. Its modular design—particularly the use of dynamic convolution and multi-level attention mechanisms—enables robust feature extraction and noise suppression across diverse visibility-degraded environments. We expect that DCNet can generalize to more another crops and agricultural datasets, as the underlying challenges of occlusion, low contrast, and illumination variability are common across these scenarios. Future work will explicitly validate DCNet on additional publicly available datasets to further strengthen claims regarding generalization.

## 6 Conclusions

This study addresses the challenge of fruit object detection under low-visibility conditions, which is crucial for intelligent orchard management and robotic harvesting. We propose a dynamic coding-based detection network (DCNet) that integrates a dynamic feature encoder, a global attention gate, an iterative feature attention module, and a cross-attention decoder. These components work together to effectively address feature degradation, background clutter, and fine-grained detail preservation in challenging agricultural environments.

Experimental results on the LVScene4K dataset, which includes multiple fruit categories (grapes, kiwis, oranges, pears, pomelos, persimmons, pumpkins, and tomatoes) captured under fog, rain, low light, and occlusion conditions, demonstrate that DCNet achieves an 86.5% mean average precision and 84.2% intersection over union. Compared with state-of-the-art baselines, DCNet improves the F1 by 3.4% and IoU by 4.3% while maintaining an inference speed of 28 FPS on an RTX 3090 GPU. These results not only prove that DCNet provides a superior balance between accuracy and efficiency but also show its potential for practical applications.

Our research contributions lie in the development of a novel framework that achieves efficient and accurate detection in complex agricultural environments. The design of DCNet considers the special challenges of low-visibility conditions and provides solutions through its innovative modular structure. Furthermore, our experimental results indicate that DCNet has better generalization capabilities when dealing with diverse agricultural scenarios, which is significant for the automation and precision of smart agriculture.

Future work will explore the integration of more heterogeneous data sources to further enhance the model’s generalization capabilities and applicability. Additionally, with advancements in technology, we plan to explore more advanced data augmentation and simulation techniques to improve the model’s adaptability and robustness. Overall, this study provides a promising direction for future automation and intelligent applications under broader conditions.

## Data Availability

The original contributions presented in the study are included in the article/supplementary material. Further inquiries can be directed to the corresponding authors.
